# Tuberculin skin test conversion and primary progressive tuberculosis disease in the first 5 years of life: a birth cohort study from Cape Town, South Africa

**DOI:** 10.1016/S2352-4642(17)30149-9

**Published:** 2018-01

**Authors:** Leonardo Martinez, David M le Roux, Whitney Barnett, Attie Stadler, Mark P Nicol, Heather J Zar

**Affiliations:** a Division of Infectious Diseases and Geographic Medicine, School of Medicine, Stanford University, Stanford, CA, USA; b Department of Epidemiology and Biostatistics, College of Public Health, University of Georgia, Athens, GA, USA; c Department of Paediatrics and Child Health, Red Cross War Memorial Children's Hospital and Medical Research Council Unit on Child and Adolescent Health, University of Cape Town, Cape Town, South Africa; d Division of Medical Microbiology, University of Cape Town, Cape Town, South Africa

## Abstract

**Background:**

Tuberculosis is a leading cause of global childhood mortality. However, the epidemiology and burden of tuberculosis in infancy is not well understood. We aimed to investigate tuberculin skin test conversion and tuberculosis in the Drakenstein Child Health study, a South African birth cohort in a community in which tuberculosis incidence is hyperendemic.

**Methods:**

In this prospective birth cohort study, we enrolled pregnant women older than 18 years who were between 20 and 28 weeks' gestation and who were attending antenatal care in a peri-urban, impoverished South African setting. We followed up their children for tuberculosis from birth until April 1, 2017, or age 5 years. All children received BCG vaccination at birth. Tuberculin skin tests were administered to children at 6, 12, 24, 36, 48, and 60 months of age, and at the time of a lower respiratory tract infection. An induration reaction of 10 mm or more was considered to be a tuberculin skin test conversion. To prevent boosting, we censored children with a reactive, negative tuberculin skin test.

**Findings:**

Among 915 mother–child pairs (201 [22%] HIV-positive mothers and two [<1%] HIV-positive children), 147 (16%) children had tuberculin skin test conversion, with increasing cumulative hazard with age (0·08 at 6 months, 0·17 at 12 months, 0·22 at 24 months, and 0·37 at age 36 months). For every 100 child-years, the incidence was 11·8 (95% CI 10·0–13·8) for tuberculin skin test conversion, 2·9 (2·4–3·7) for all diagnosed tuberculosis, and 0·7 (0·4–1·0) for microbiologically confirmed tuberculosis. Isoniazid preventive therapy was effective in averting disease progression (adjusted hazard ratio 0·22, 95% CI 0·08–0·63; p<0·0001). Children with a lower respiratory tract infection were significantly more likely to also have tuberculosis than were those without one (2·27, 1·42–3·62; p<0·0001).

**Interpretation:**

Greater focus should be placed on the first years of life as a period of high burden of transmission and clinical expression of tuberculosis infection and disease. Multifaceted interventions, such as isoniazid preventive therapy and tuberculosis screening of infants with LRTIs, beginning early in life, are needed in high-burden settings.

**Funding:**

Bill & Melinda Gates Foundation, Medical Research Council South Africa, and National Research Foundation South Africa.

## Introduction

Tuberculosis is a leading cause of child mortality worldwide and most of these deaths occur before age 5 years.^1^ Approximately 1 million children, half of them younger than 5 years, develop the disease every year.^2^, ^3^ One in five children with untreated tuberculosis die of the disease,^4^, ^5^ but still more than 65% of children with tuberculosis in high-burden settings remain undiagnosed and untreated.^2^, ^3^ In sub-Saharan Africa, children represent a disproportionately high proportion of all tuberculosis cases^2^, ^6^ because of a young population age structure, high force of infection, and a raised risk of rapid progressive disease compared with adults. In South Africa, 15–30% of all tuberculosis cases probably occur in children.^3^, ^7^, ^8^ A greater understanding of the incidence and risk factors of tuberculosis infection and disease is thus necessary to develop pragmatic interventions for young children in high-burden areas.

Detecting new cases of tuberculosis in infants and young children from resource-constrained settings is difficult because childhood tuberculosis surveillance is poor,^8^ diagnostic tests for childhood tuberculosis are insensitive,^9^ and availability of testing tools and clinical training is restricted.^10^ In HIV-endemic settings, such as sub-Saharan Africa, distinct clinical presentations due to HIV can present additional diagnostic challenges.^11^, ^12^ Furthermore, most estimates of disease incidence are based on data from health-care facilities and do not reflect true community-based incidence. Because of these difficulties, few studies have systematically and prospectively investigated the community burden of tuberculosis in children, or prenatal and early-life risk factors for the development of tuberculosis infection and disease.

**Research in context**

**Evidence before this study**

We searched PubMed twice for articles published, in English, up to July 31, 2017. The search terms used were “(child* OR pediatric OR infant) AND (tuberculosis OR TB) AND (incidence)” for the first search and “(‘birth cohort’) AND (tuberculosis OR TB)” for the second search. Both searches were restricted to the title and abstract fields. We found few community-based studies that investigated the incidence of tuberculosis-related outcomes in young children. A study from South Africa reported a Quantiferon Gold In-Tube conversion rate in infants of 7% after 1 year and incident tuberculosis of 2% measured over the subsequent 6–24 months. Accurate longitudinal data from high-burden settings are scarce for the incidence of *Mycobacterium tuberculosis* infection and primary progressive disease in infants and young children, and for prenatal and early-life risk factors for tuberculosis-related outcomes.

**Added value of this study**

To our knowledge, this is the first community-based prospective birth cohort study to investigate the incidence of and risk factors for *M tuberculosis* infection and primary progressive disease during the first 5 years of life. We noted a high incidence of tuberculin skin test conversion and primary progressive disease, and this was especially apparent before age 2 years. We also identified maternal smoking during pregnancy and lower respiratory tract infection as novel risk factors for paediatric tuberculosis, and male sex as a risk factor for tuberculin conversion.

**Implications of all the available evidence**

Results from this South African birth cohort study indicate that greater focus should be placed on the first years of life as a period of high burden of transmission and clinical expression of tuberculosis. The high rates of tuberculosis-related outcomes are alarming and suggest a considerable burden of unidentified transmission and undiagnosed tuberculosis disease among infants and young children in high-burden settings. There is an urgent need to review global guidelines on the management of paediatric tuberculosis in high-burden settings. Re-examination of guidelines should include consideration of integrating paediatric lower respiratory tract infection and tuberculosis control programmes. Tuberculosis infection and disease screening when infants present to primary care clinics with lower respiratory tract infections could identify a high number of undiagnosed paediatric cases. Scale-up of preventive therapy to infants with primary infection or at high risk for disease progression, such as tuberculosis-exposed or HIV-infected children, is essential to help decrease childhood mortality in sub-Saharan Africa.

We aimed to investigate tuberculin skin test conversion and tuberculosis in infants and young children from the Drakenstein Child Health study, a South African birth cohort in a community with a high tuberculosis burden.^13^

## Methods

### Study design and participants

In this prospective birth cohort study, we enrolled pregnant women who were between 20 and 28 weeks' gestation and attending antenatal care in Paarl, a peri-urban setting outside of Cape Town, South Africa.^14^ In 2015, tuberculosis incidence in this area was estimated to be 880 new cases per 100 000 population.^13^ Participants were recruited from two clinics, TC Newman and Mbekweni, which are a few miles apart. Both clinics serve impoverished, heterogeneous communities. People attending TC Newman are of admixed ancestry, whereas the Mbekweni clinic serves mostly a black, Xhosa population. All infants were given BCG vaccination at birth, per national policy. All mothers accessed care in the public sector, which has a strong primary health-care programme, including an effective mother-to-child HIV prevention and antiretroviral therapy programme.

Women were followed up through pregnancy and childbirth, and newborn infants were followed up into early childhood, up to age 5 years. Exclusion criteria for pregnant women were being younger than 18 years and intending to leave the area within 1 year.

We obtained ethics approval from the University of Cape Town Faculty of Health Sciences Human Research Ethics Committee (reference numbers 401/2009 and 651/2013) and the Provincial Child Health Research Committee. Mothers provided written informed consent at enrolment and verbal assent for infants, which was renewed annually.

### Procedures

Comprehensive questionnaires about maternal health were administered at enrolment and antenatal data were collected concurrently. Detailed birth information was obtained at delivery. Obstetric care and all births took place at the regional hospital in Paarl. Follow-up visits, including clinical examinations, were done at 6, 12, 24, 36, 48, and 60 months of age. Data for environmental exposures, household characteristics, respiratory risk factors, anthropometry, and child symptoms were obtained at scheduled visits. Missed visits were rebooked with a study mobile phone network system or by study community-based fieldworkers. Mothers were counselled about respiratory symptoms at every visit and advised to attend the study site or contact study staff between scheduled study visits whenever the child developed cough or difficulty breathing. Socioeconomic status comprised a comprehensive composite of asset ownership, household income, employment, and education.^14^

HIV tests were given to all mothers during pregnancy. Adults were tested with Abbott Determine HIV 1/2 rapid HIV antibody test (Abbott Laboratories, North Chicago, IL, USA). If positive, a confirmatory enzyme-linked immunosorbent assay was done. Infants of HIV-positive mothers were tested with DNA PCR (Cobas Ampliprep system, Roche Molecular Systems, Branchburg, NJ, USA) at age 6 weeks, and 6 weeks after the end of breastfeeding. Children were re-tested at 18 months with the rapid antibody test.

As part of the Drakenstein Child Health Study, we established surveillance systems for the detection of lower respiratory tract infections in infants; children with such infections were seen and had specimens (induced sputum, nasopharyngeal samples, blood, urine [all participants], and blood culture [hospitalised infants with lower respiratory tract infections]) taken by trained study nurses and staff.^14^, ^15^ Briefly, study nurses were trained to diagnose lower respiratory tract infection or severe lower respiratory tract infection according to WHO clinical case definitions.^16^ A lower respiratory tract infection was diagnosed in children with cough or difficulty breathing and age-specific tachypnoea, or if the child had lower-chest-wall indrawing. Severe lower respiratory tract infections were diagnosed in children younger than 2 months with tachypnoea or lower-chest-wall indrawing, or in children of any age if the child had a general danger sign.

We derived *Z* scores from WHO child growth standards at birth and at every follow-up visit, and we used the median of all the weight-for-age *Z* scores for each child to summarise nutrition status during the follow-up period. Children were considered to be severely underweight or stunted if weight-for-age or length-for-age *Z* scores were less than −2. Normal weight was −2 to 2, and overweight was a score greater than 2.

Tuberculin skin tests were done at the 6-month visit and then at 12, 24, 36, 48, and 60 months of age, and at the time of a lower respiratory tract infection. Tuberculin skin test conversion was defined as an induration reaction greater than or equal to 10 mm, to minimise the risk of misclassification due to BCG vaccination or exposure to environmental mycobacteria, as recommended by WHO and South Africa's Department of Health.^10^, ^17^ To prevent misinterpretation of boosted skin-test reactions due to recurrent tuberculin skin testing as tuberculosis infection, children with a reactive but negative skin test (1–9 mm) were not given another test, and were censored for the tuberculin skin test conversion analysis at that point in time. Because of the high number of censored skin tests before age 48 months, we excluded tuberculin skin tests taken after 36 months of age. Children with positive skin tests were referred to local tuberculosis clinics for isoniazid preventive therapy; however, the study investigators could not enforce that this was prescribed.

Children were followed up for tuberculosis from birth until April 1, 2017, or age 5 years. Trained study staff collected induced sputum specimens in duplicate for tuberculosis culture and mycobacterial PCR investigation (Xpert MTB/RIF; Cepheid, Sunnyvale, CA, USA) from all children with a tuberculin skin test induration of at least 10 mm, and from children who were suspected to have or had been diagnosed with tuberculosis by local health services. A chest radiograph was taken in all children with suspected pulmonary tuberculosis. Tuberculosis was diagnosed by experienced physicians and nurses in local tuberculosis community clinics, and chest radiographs were read and reported by an experienced tuberculosis clinician. We compared results using three different definitions of tuberculosis: all tuberculosis cases (clinically, radiographically suggestive, or microbiologically confirmed cases); cases that were microbiologically confirmed or radiographically suggestive only; and microbiologically confirmed cases only (positive Xpert MTB/RIF or sputum culture).

### Statistical analysis

Mother–child pairs were included in this analysis if they had at least one tuberculin skin test. For exploratory data analysis, we summarised continuous variables as medians with IQRs, and categorical variables using proportions.

For tuberculin skin test conversion, time-to-event was determined by the date on which the child was administered a skin test and had a positive result; a child was determined to have no conversion on the date of the last negative skin test. Follow-up was censored at a reactive skin test, death, or age 3 years. For tuberculosis, time-to-event was determined when a child was diagnosed with tuberculosis. Follow-up was censored at death, 5 years of age, or April 1, 2017. Children with reactive but negative tuberculin skin tests were not censored for the tuberculosis survival analysis. We analysed factors associated with tuberculin skin test conversion and tuberculosis for the whole cohort and adjusted for the enrolment site for each analysis. Because the first tuberculin skin test was administered at different ages (because of missed study visits or lower respiratory tract infections), we also adjusted for the age of first tuberculin skin test administration in the multivariable model of skin test conversion. We used Cox proportional hazard models for multivariable modelling, including a random intercept for each child using a gamma distribution, and results are presented as hazard ratios (HRs). For each independent variable, we present univariable models (indirect effects) and multivariable models (direct effects, after adjustment for the effects of confounding variables).

We explored the effect of the censoring approach used in our tuberculin skin test conversion analysis by adjusting our definition of conversion to infants with induration reactions more than 5 mm (rather than the 10 mm cutoff used in our primary conversion analysis). In this analysis, all children with reactive but negative tuberculin induration reactions less than 5 mm were censored.

We investigated the timing of lower respiratory tract infections and tuberculosis diagnoses to determine whether case ascertainment bias could possibly explain an association seen between the two diseases. We assumed case ascertainment was likely if diagnosis of both diseases occurred simultaneously in children with multiple diagnoses. To investigate this, among children diagnosed with both diseases during the study period, we calculated the proportion diagnosed with a lower respiratory tract infection and tuberculosis less than 2 weeks apart.

We used two-sided p values and 95% CIs to assess statistical significance in all models. The likelihood ratio test was used to derive all p values from Cox regression models. We did all analyses with Stata (version 14.1).

### Role of the funding source

The funders of the study had no role in study design, data collection, data analysis, data interpretation, or writing of the report. All authors had full access to all the data in the study and had final responsibility for the decision to submit for publication.

## Results

**Figure 1:**
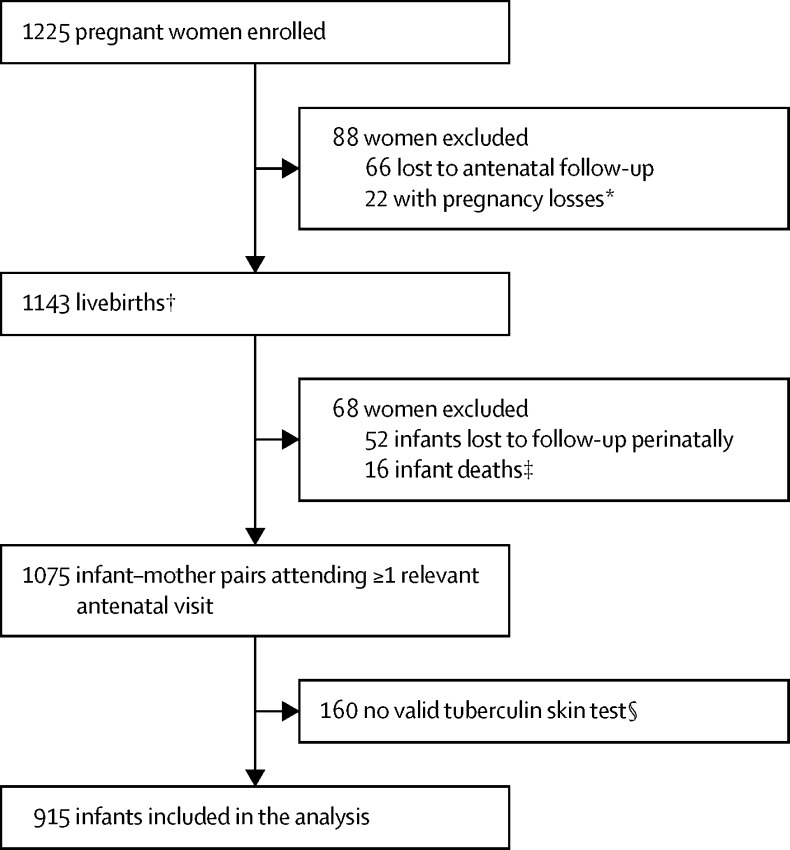
Study flow *Loss of pregnancy due to miscarriage, stillbirth, or intrauterine death (23 infants [including one set of twins]). †Including four pairs of twins and one set of triplets. ‡No postnatal data collected. §Participants did not have a valid tuberculin skin test result, most commonly because of national tuberculin shortages, but also because mothers and infants did not return for the induration reading.

Between March 5, 2012, and March 31, 2015, 1225 pregnant women were recruited and enrolled in the birth cohort (figure 1). Of 1143 livebirths, 68 (6%) were excluded because of perinatal death or study termination, and 160 infants (14%) were enrolled but did not have a valid tuberculin skin test, predominantly because of national and global shortages of tuberculin. Thus, 915 infants with tuberculin skin test results and follow-up for active disease were included in our analysis. The characteristics of excluded infants were largely similar to those of included infants, except that mothers of excluded infants were more likely to be young, have a high income, and were less likely to have ever been previously diagnosed with tuberculosis (appendix).

Although 201 (22%) mothers were HIV-seropositive, only two (<1%) infants tested were HIV-positive, because of an effective antenatal antiretroviral therapy programme (table 1). These HIV-positive infants were breastfed but did not have household tuberculosis exposure (appendix). 850 (93%) women breastfed their newborn infants. Household tuberculosis exposure in the year before the primary study visit occurred in 128 (14%) of all surveyed households, and was almost twice as frequent at Mbekweni than at TC Newman (18% *vs* 10%, p<0·003). 440 (48%) of 915 infants had at least one lower respiratory tract infection; of 440 cases, 100 (23%) led to hospital admission and 101 (23%) were classified as severe.

**Table 1:** Sociodemographic and clinical characteristics of included mother–child pairs, by clinic

		**TC Newman (n=420)**	**Mbekweni (n=495)**	**Total (n=915)**
**Infant characteristics**				
Sex				
	Male	230 (55%)	238 (48%)	468 (51%)
	Female	190 (45%)	257 (52%)	447 (49%)
Birthweight (kg)		3·0 (2·6 to 3·4)	3·2 (2·8 to 3·5)	3·1 (2·7 to 3·4)
Low birthweight (<2·5 kg)		72 (17%)	63 (13%)	135 (15%)
Gestational age (weeks)		39 (38 to 40)	39 (38 to 40)	39 (38 to 40)
Preterm birth (<37 weeks)		60 (14%)	87 (18%)	147 (16%)
Lower respiratory tract infection		198 (47%)	242 (49%)	440 (48%)
Severe lower respiratory tract infection		37 (9%)	64 (13%)	101 (11%)
Hospitalised for lower respiratory tract infection		45 (11%)	55 (11%)	100 (11%)
HIV-positive		0	2 (<1%)	2 (<1%)
HIV-exposed		14 (3%)	187 (38%)	201 (22%)
Breastfed		419 (100%)	431 (87%)	850 (93%)
Weight-for-age *Z* score		−0·75 (−1·36 to −0·06)	−0·40 (−1·23 to 0·31)	−0·54 (−1·31 to 0·10)
Weight-for-age *Z* score				
	Underweight	46 (11%)	38 (8%)	84 (9%)
	Normal weight	370 (88%)	442 (89%)	812 (89%)
	Overweight	3 (1%)	12 (2%)	15 (2%)
	Missing	1 (<1%)	3 (1%)	4 (<1%)
Isoniazid preventive therapy		31 (7%)	30 (6%)	61 (7%)
**Maternal characteristics**				
Age (years)		24·9 (21·4 to 29·7)	27·2 (22·6 to 32·2)	26·3 (22·1 to 31·0)
Married or cohabitating		180 (43%)	186 (38%)	366 (40%)
Tuberculosis treatment during pregnancy		20 (5%)	21 (4%)	41 (4%)
Ever diagnosed with tuberculosis before pregnancy		13 (3%)	25 (5%)	38 (4%)
Maternal smoking during pregnancy*		195 (46%)	23 (5%)	218 (24%)
Maternal education				
	Primary school only	33 (8%)	40 (8%)	73 (8%)
	Some secondary school	225 (54%)	275 (56%)	500 (55%)
	Finished secondary school	162 (39%)	180 (36%)	342 (37%)
Formal employment		123 (29%)	120 (24%)	243 (27%)
**Household characteristics**				
Socioeconomic status (quartile)				
	Lowest	77 (18%)	156 (32%)	233 (25%)
	Moderate low	110 (26%)	132 (27%)	242 (26%)
	Moderate high	112 (27%)	114 (23%)	226 (25%)
	Highest	114 (27%)	92 (19%)	206 (23%)
	Missing data	7 (2%)	1 (<1%)	8 (1%)
Household income (rand per month)				
	<1000	144 (34%)	210 (42%)	354 (39%)
	1000–5000	210 (50%)	232 (47%)	442 (48%)
	>5000	66 (16%)	48 (10%)	114 (12%)
Housing				
	Shack or hokkie	124 (30%)	202 (41%)	326 (36%)
	House or flat	296 (70%)	293 (59%)	589 (64%)
Crowding (people per household)		5 (4 to 7)	4 (3 to 6)	4 (3 to 6)
	≤3	92 (22%)	205 (41%)	297 (32%)
	4–5	169 (40%)	144 (29%)	313 (34%)
	>5	157 (37%)	146 (29%)	303 (33%)
	Missing data	2 (1%)	0	2 (<1%)
Children younger than 5 years per household				
	0	252 (60%)	311 (63%)	563 (62%)
	1	139 (33%)	152 (31%)	291 (32%)
	>1	29 (7%)	32 (6%)	61 (7%)
Household exposure to tuberculosis in the year before first study visit		40 (10%)	88 (18%)	128 (14%)

Data are n (%) or median (IQR). Column totals vary across different characteristics because of missing values for some participants.

* Self-reported smoking status at the baseline study visit; self-reported smoking was highly correlated with maternal cotinine levels.

**Figure 2:**
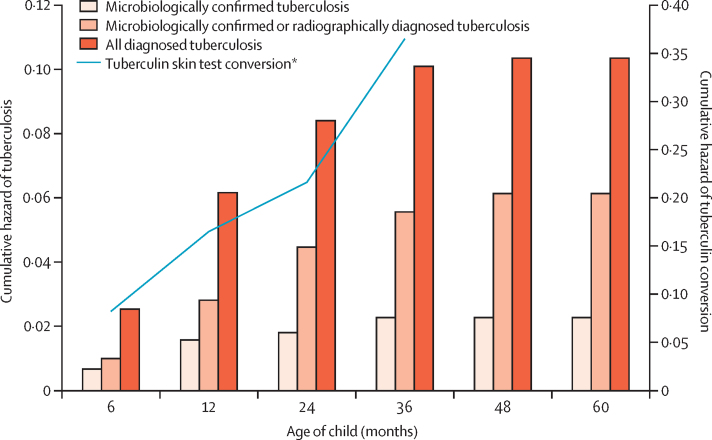
Cumulative risk of paediatric tuberculosis and tuberculin skin test conversion *Analysed for the first 3 years of life.

For the tuberculin skin test conversion analysis, 915 children accrued 1248·7 years of follow-up (table 2). In total, 147 children (16%) had a positive result and most of these conversions occurred before age 1 year (appendix). All of these children were referred to local clinics for isoniazid preventive therapy, but only 33 (22%) were medically registered as receiving the treatment. The incidence of tuberculin skin test conversion was 11·8 (95% CI 10·0–13·8) per 100 child-years. The cumulative hazard of conversion was high 6 months after birth (0·08) and consistently increased with age to 0·17 after 12 months, 0·22 after 24 months, and 0·37 after 36 months (figure 2; appendix).

**Table 2:** Tuberculin skin test conversion in children, stratified by child, maternal, and household characteristics

			**Tuberculin conversion events (n)**	**Partici-pants (n)**	**Child-years of obser-vation**	**Incidence per 100 child-years (95% CI)**	**HR (95% CI, p value)**
All participants			147	915	1248·7	11·8 (10·0–13·8)	··
Child characteristics							
	Sex						
		Female	61	447	647·7	9·4 (7·3–12·1)	1 (ref)
		Male	86	468	600·1	14·3 (11·6–17·7)	1·53 (1·10–2·13, p=0·011)
	Birthweight*						
		Normal	126	780	1071·3	11·8 (9·9 −14·0)	1 (ref)
		Low	21	135	176·5	11·9 (7·8–18·2)	1·00 (0·63–1·58, p=0·99)
	Gestational age†						
		Full term	124	768	1042·3	11·9 (10·0–14·2)	1 (ref)
		Preterm	23	147	205·5	11·2 (7·4–16·8)	0·93 (0·60–1·45, p=0·75)
	Lower respiratory tract infection						
		No	74	475	660·4	11·2 (8·9–14·1)	1 (ref)
		Yes	73	440	587·4	12·4 (9·9–15·6)	1·12 (0·81–1·54, p=0·51)
	HIV-positive						
		No	147	913	1246·0	11·8 (10·0–13·9)	··
		Yes	0	2	1·8	··	··
	Weight-for-age *Z* score‡						
		Underweight	11	84	101·2	10·9 (6·0 −19·6)	0·86 (0·46–1·58, p=0·62)
		Normal weight	134	812	1111·4	12·1 (10·2–14·3)	1 (ref)
		Overweight	1	15	27·0	3·7 (0·5–26·3)	0·31 (0·44–2·24, p=0·25)
	Feeding choice						
		Did not breastfeed	5	65	85·6	5·8 (2·4–14·0)	1 (ref)
		Breastfed	142	850	1162·2	12·2 (10·4–14·4)	1·98 (0·81–4·83, p=0·13)
Maternal characteristics							
	Age (years)		··	··	··	··	0·98 (0·95–1·01, p=0·11)
	Tuberculosis treatment during pregnancy						
		No	138	874	1194·2	11·6 (9·8–13·7)	1 (ref)
		Yes	9	41	53·6	16·8 (8·7–32·3)	1·47 (0·75–2·89, p=0·26)
	Ever diagnosed with tuberculosis‡						
		No	146	874	1190·4	12·3 (10·4–14·4)	1 (ref)
		Yes	1	38	50·5	2·0 (0·3–14·0)	0·15 (0·02–1·11, p=0·063)
	Maternal smoking during pregnancy‡§						
		No	103	692	953·2	10·8 (8·9–13·1)	1 (ref)
		Yes	43	218	286·1	15·0 (11·1–20·3)	1·37 (0·96–1·95, p=0·086)
	Maternal education						
		Primary school only	15	73	102·8	14·6 (8·0–24·2)	1 (ref)
		Some secondary school	73	500	676·6	10·8 (8·6–13·6)	0·73 (0·42–1·27, p=0·26)
		Finished secondary school	59	342	468·4	12·6 (9·8–16·3)	0·86 (0·49–1·51, p=0·60)
Household characteristics							
	Socioeconomic status (quartile)‡						
		Highest	35	206	284·9	12·3 (8·8–17·1)	1 (ref)
		Lowest	45	233	313·7	14·3 (10·7–19·2)	1·14 (0·73–1·77, p=0·56)
		Moderate low	32	242	331·9	9·6 (6·8–13·6)	0·82 (0·48–1·25, p=0·30)
		Moderate high	35	226	303·8	11·5 (8·3–16·0)	0·93 (0·58–1·48, p=0·75)
	Clinic						
		Mbekweni	72	495	681·6	10·6 (8·4–13·3)	1 (ref)
		TC Newman	75	420	566·2	13·2 (10·6–16·6)	1·24 (0·90–1·71, p=0·19)
	Household income (rand per month)						
		>5000	16	114	166·3	9·6 (5·9–15·7)	1 (ref)
		1000–5000	69	442	627·1	11·0 (8·7–13·9)	1·07 (0·62–1·85, p=0·80)
		<1000	62	354	454·4	13·6 (10·6–17·5)	1·34 (0·77–2·32, p=0·30)
	Crowding (people per household)‡						
		≤3	44	297	412·4	10·7 (7·9–14·3)	1 (ref)
		4–5	41	313	438·3	9·4 (6·9–12·7)	0·86 (0·56–1·32, p=0·49)
		>5	62	303	396·1	15·7 (12·2–20·1)	1·43 (0·97–2·11, p=0·067)
	Children younger than 5 years per household						
		0	80	563	776·6	10·3 (8·3–12·8)	1 (ref)
		1	54	291	406·0	13·3 (10·2–17·4)	1·31 (0·93–1·85, p=0·13)
		>1	13	61	65·3	19·9 (11·6–34·3)	1·81 (1·00–3·25, p=0·048)
	Patient with tuberculosis in household in past year‡						
		No	131	779	1075·5	12·2 (10·3–14·5)	1 (ref)
		Yes	13	128	162·6	8·0 (4·6–13·8)	0·65 (0·37–1·15, p=0·14)

Missing data are detailed in table 1. HR=hazard ratio.

* Normal birthweight is 2·5 kg or more and low birthweight is less than 2·5 kg.

† Full term is 37 weeks or more and preterm is less than 37 weeks.

‡ Some data are missing for this variable.

§ Self-reported smoking status at the baseline study visit; self-reported smoking was highly correlated with maternal cotinine levels.

Univariable Cox regression of tuberculin conversion and tuberculosis results is shown in Table 2, Table 3. In univariable analyses, boys and children in households with more than one child younger than 5 years had significantly increased risk of tuberculin skin test conversion (table 2). A larger proportion of infants exposed to maternal smoking during pregnancy had tuberculin conversion, but the HR was not significant. After adjustment for potential confounders in a multivariable model, male sex (adjusted HR 1·54, 95% CI 1·11–2·15; p=0·010; table 4) was the only significant predictor of increased tuberculin skin test conversion. In a sensitivity analysis using a 5 mm cutoff to define tuberculin conversion, the incidence of tuberculin conversion per 100 child-years increased to 20·0 (95% CI 17·6–22·7).

**Table 3:** Diagnosed tuberculosis in children during follow-up, stratified by child, maternal, and household characteristics

				Disease events*(n)	**Partici-pants (n)**	**Child-years of obser-vation**	**Incidence per 100 child-years (95% CI)**	**HR (95% CI, p value)**
All participants				81	915	2736·8	2·9 (2·4–3·7)	··
Child characteristics								
	Sex							
		Female		32	447	1355·3	2·4 (1·7–3·3)	1 (ref)
		Male		49	468	1381·5	3·5 (2·7 − 4·7)	1·50 (0·96–2·35, p=0·073)
	Birthweight†							
			Normal	64	780	2331·1	2·7 (2·1–3·5)	1 (ref)
			Low	17	135	405·8	4·2 (2·6–6·7)	1·56 (0·91–2·66, p=0·10)
	Gestational age‡							
		Full term		65	768	2298·0	2·8 (2·2 − 3·6)	1 (ref)
		Preterm		16	147	438·8	3·6 (2·2 − 6·0)	1·30 (0·76–2·25, p=0·34)
	Lower respiratory tract infection							
		No		27	475	1502·0	1·8 (1·2 − 2·6)	1 (ref)
		Yes		54	440	1234·9	4·4 (3·3 − 5·7)	2·29 (1·44–1·64, p<0·0001)
	HIV status							
		Negative		80	913	2731·8	2·9 (2·4 − 3·6)	1 (ref)
		Positive		1	2	5·1	19·8 (2·8 − 100·0)	6·42 (0·89–46·14, p=0·065)
	Weight-for-age *Z* score§							
		Underweight		7	84	244·6	2·9 (1·4 − 6·0)	0·94 (0·43–2·04, p=0·88)
		Normal weight		73	812	2437·9	3·0 (2·4 − 3·8)	1 (ref)
		Overweight		1	15	41·1	2·4 (0·3 − 17·3)	0·75 (0·10–5·43, p=0·78)
	Breastfeeding							
		Did not breastfeed		2	65	202·4	1·0 (0·2 −4·0)	1 (ref)
		Breastfed		79	850	2534·4	3·1 (2·5–3·9)	3·26 (0·80–13·36, p=0·10)
	Isoniazid preventive therapy							
		Among converters						
			No	43	117	261·6	16·4 (12·2–22·2)	1 (ref)
			Yes	2	33	110·1	1·8 (0·0–7·3)	0·13 (0·03–0·56, p=0·0058)
		Among non-converters						
			No	33	737	2271·1	1·5 (1·0–2·0)	1 (ref)
			Yes	3	28	94·1	3·2 (1·0–9·9)	2·36 (0·72–7·67, p=0·16)
Maternal characteristics								
	Age (years)			··	··	··	··	0·98 (0·94–1·02, p=0·37)
	Tuberculosis treatment in pregnancy							
		No		75	874	2627·0	3·0 (2·3–3·6)	1 (ref)
		Yes		6	41	109·8	5·5 (2·5–12·2)	1·78 (0·78–4·09, p=0·17)
	Ever diagnosed with tuberculosis§							
		No		79	874	2619·3	3·0 (2·4–3·8)	1 (ref)
		Yes		2	38	109·0	1·8 (0·4–7·3)	0·59 (0·14–2·39, p=0·46)
	Maternal smoking during pregnancy§¶							
		No		46	692	2097·4	2·2 (1·6–2·9)	1 (ref)
		Yes		34	218	622·6	5·5 (3·9–7·6)	2·47 (1·58–3·85, p<0·0001)
	Maternal education§							
		Primary school only		10	73	218·4	4·6 (2·5–8·5)	1 (ref)
		Some secondary school		45	500	1486·4	3·0 (2·3–4·1)	0·65 (0·33–1·29, p=0·22)
		Finished secondary school		26	342	1032·0	2·5 (1·7–3·7)	0·55 (0·26–1·13, p=0·11)
Household characteristics								
	Socioeconomic status (quartile)§							
		Lowest		24	233	726·6	3·3 (2·2–4·9)	1 (ref)
		Moderate low		23	242	712·2	3·2 (2·1–4·9)	0·94 (0·53–2·42, p=0·83)
		Moderate high		18	226	656·6	2·7 (1·7–4·4)	0·79 (0·43–1·46, p=0·46)
		Highest		15	206	614·9	2·4 (1·5–4·0)	0·72 (0·38–1·37, p=0·32)
	Clinic							
		Mbekweni		26	495	1472·4	1·8 (1·2–2·6)	1 (ref)
		TC Newman		55	420	1264·4	4·3 (3·3–5·7)	2·55 (1·60–4·07, p<0·0001)
	Household income (rand per month)							
		<1000		38	354	1114·1	3·4 (2·5–4·7)	1 (ref)
		1000–5000		38	442	1261·2	3·0 (2·2–4·1)	0·84 (0·53–1·31, p=0·43)
		>5000		5	114	361·5	1·4 (0·6–3·3)	0·41 (0·16–1·03, p=0·059)
	Crowding (people per household)§							
		≤3		18	297	886·3	2·0 (1·3–3·2)	1 (ref)
		4–5		28	313	940·6	3·0 (2·1–4·3)	1·50 (0·83–2·70, p=0·18)
		>5		35	303	904·2	3·9 (2·8–5·4)	1·94 (1·10–3·42, p=0·022)
	Children younger than 5 years per household							
		0		42	563	1705·4	2·5 (1·8–3·3)	1 (ref)
		1		31	291	851·5	3·6 (2·6–5·2)	1·45 (0·91–2·31, p=0·12)
		>1		8	61	179·9	4·4 (2·2–8·0)	1·75 (0·82–3·73, p=0·15)
	Patient with tuberculosis in household in past year§							
		No		64	779	2386·1	2·7 (2·1–3·4)	1 (ref)
		Yes		15	128	329·5	4·6 (2·7–7·6)	1·52 (0·86–2·67, p=0·15)

Missing data are detailed in table 1. HR=hazard ratio.

* Any tuberculosis diagnosis made in the clinic (clinically, radiographically, or microbiologically).

† Normal birthweight is 2·5 kg or more and low birthweight is less than 2·5 kg.

‡ Full term is 37 weeks or more and preterm is less than 37 weeks.

§ Some data are missing for this variable.

¶ Self-reported smoking status at the baseline study visit; self-reported smoking was highly correlated with maternal cotinine levels

**Table 4:** Risk factors for tuberculin skin test conversion and tuberculosis in multivariable model

		**Tuberculin skin test conversion**	**All diagnosed tuberculosis**	**Microbiologically confirmed or radiographically suggestive tuberculosis**	**Microbiologically confirmed tuberculosis**
Male sex		1·54 (1·11–2·15; p=0·010)	1·15 (0·73–1·81); p=0·53	··	··
Maternal smoking during pregnancy*		1·13 (0·75–1·71; p=0·55)	1·69 (1·02–2·79); p=0·040	1·07 (0·50–2·25); p=0·87	··
Isoniazid preventive therapy		NA	0·22 (0·08–0·63), p<0·0001	0·28 (0·07–1·21); p=0·084	··
Tuberculin skin test conversion		NA	9·44 (6·01–14·82); p<0·0001	5·57 (3·03–10·22); p<0·0001	2·29 (0·81–6·43); p=0·12
Lower respiratory tract infection†		··	2·27 (1·42–3·62), p<0·0001	3·11 (1·60–6·03); p=0·0008	9·06 (2·08–39·42); p=0·0033
Children younger than 5 years in household‡					
	1	1·23 (0·87–1·75; p=0·12)	··	··	··
	>1	1·62 (0·90–2·92; p=0·11)	··	··	··

Data are adjusted hazard ratios (95% CI; p value). We adjusted all models for the site of enrolment in addition to all included characteristics. The multivariable model of tuberculin skin test conversion was also adjusted for the age at which the first tuberculin skin test was done. NA=not applicable.

* Self-reported smoking status at the baseline study visit; self-reported smoking was highly correlated with maternal and infant urine cotinine levels.

† Diagnosed during the first 5 years of life.

‡ Reference category was no children younger than 5 years in the household.

For tuberculosis, 2736·8 child-years of follow-up were accrued (table 3). 81 children (9%) were diagnosed with tuberculosis, of whom 45 had microbiologically confirmed or radiographically suggestive disease, and 18 of such cases were microbiologically confirmed. Of the 18 microbiologically confirmed cases, 15 (83%) were symptomatic and included chronic cough, weight loss (or failure to thrive), strong contact history, a suggestive chest radiograph examination, or an acute lower respiratory tract infection. Two infants were further investigated with a microbiological exam because of tuberculin skin test conversion alone, while one was investigated because of a tuberculin skin test conversion and a confirmed close household contact.

One infant was diagnosed and treated for tuberculosis meningitis, and two were resistant to rifampin. Per 100 child-years, the incidence of all diagnosed tuberculosis was 2·9 (95% CI 2·4–3·7), incidence of microbiologically confirmed or radiographically suggestive tuberculosis was 1·6 (95% CI 1·2–2·2), and incidence of microbiologically confirmed tuberculosis was 0·7 (95% CI 0·4–1·0). Cumulative hazard at the end of follow-up was 0·10 for all diagnosed disease and 0·02 for microbiologically confirmed disease, and increased most in the first 3 years of life (figure 2; appendix). Among participants who had a tuberculin skin test conversion, the hazard of diagnosed paediatric tuberculosis was approximately 0·4 in infancy (<1 year old) and decreased substantially to less than 0·1 by age 2 years.

**Figure 3:**
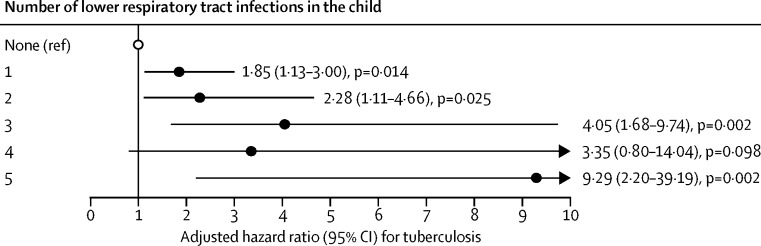
Adjusted hazard ratios for tuberculosis by number of lower respiratory tract infections in the child Data were analysed using Cox proportional hazard modelling and adjusted for the enrolment site.

In the univariable analysis, risk factors for diagnosed tuberculosis included male sex, maternal smoking during pregnancy, TC Newman clinic, households with more than five people, and a lower respiratory tract infection (table 3). Tuberculosis risk increased with the number of lower respiratory tract infections, regardless of the diagnostic definition used for tuberculosis (p_trend_<0·0001; figure 3; appendix). The Cox model was identifiable after applying the random effects, and the proportional hazards assumption was met for all Cox regression analyses of both outcomes, except for socioeconomic status (both outcomes) and crowding for tuberculosis (appendix).

In multivariable analysis, risk factors for diagnosed tuberculosis that remained significant were tuberculin skin test conversion, maternal smoking during pregnancy, and a lower respiratory tract infection. Isoniazid preventive therapy was also effective in averting disease progression (table 4). When we confined tuberculosis definition to radiographically suggestive or microbiologically confirmed disease, tuberculin skin test conversion and a lower respiratory tract infection remained significant predictive factors (table 4). The protective effect of isoniazid preventive therapy had a similar point estimate to that noted for all diagnosed tuberculosis cases, although the confidence intervals were wider (table 4). When including only microbiologically confirmed tuberculosis, the only factor that remained associated with tuberculosis was a lower respiratory tract infection (table 4). When restricting the analysis to only the TC Newman clinic, infants exposed to maternal smoking during pregnancy were at a higher risk of diagnosed tuberculosis, but this did not reach significance (adjusted HR 1·66, 0·96–2·86; appendix).

To explore the potential role of case ascertainment in determining the relation between tuberculosis and lower respiratory tract infections, we investigated the timing of a lower respiratory tract infection in relation to tuberculosis for each child with both diagnoses (n=55; appendix). In most cases, both diagnoses occurred at least 2 weeks apart, regardless of the method used to ascertain tuberculosis (41 [75%] of 55 with diagnosed tuberculosis, 26 [76%] of 34 with radiographically suggestive or microbiologically confirmed diagnoses, and 11 [65%] of 17 with microbiologically confirmed disease). Moreover, the diagnosis of tuberculosis occurred before that of the lower respiratory tract infection in 23 (42%) of 55 diagnosed tuberculosis cases, 16 (47%) of 34 radiographically suggestive or microbiologically confirmed diagnoses, and six (35%) of 17 microbiologically confirmed disease cases. It is thus unlikely that the association is due only to case ascertainment.

## Discussion

Few prospective studies have measured community-based incidence and prenatal and early-life risk factors for tuberculosis transmission and disease in high-burden areas. As a result, pragmatic approaches to guide prevention and management strategies for young children in these settings are absent. In this longitudinal birth cohort study of 915 infants and young children from South Africa, we noted a high burden of *Mycobacterium tuberculosis* infection and subsequent disease. The incidence for tuberculin skin test conversion and primary progressive disease found in our study—equating to 2900 cases per 100 000 population per year—is alarming and suggests a considerable burden of unidentified transmission and undiagnosed tuberculosis among young children in this community. We also identified modifiable risk factors for these outcomes that have important practical implications, and which could be used to reduce infections and prevent incident tuberculosis in children.

Incidences of both tuberculosis infection and disease in our cohort are among the highest reported in young children and are slightly higher than those reported in a study of South African infants enrolled in a vaccine trial.^18^ In that study, Quantiferon Gold In-Tube conversion after approximately 1 year was 7%, and incident tuberculosis was 2% for diagnosed disease (2% for microbiologically confirmed tuberculosis) measured over the subsequent 6–24 months. Although clinical overdiagnosis might have occurred in our study, we believe this is unlikely for two reasons. First, despite the low sensitivity of Xpert and sputum culture testing in young children, the incidence of microbiologically confirmed tuberculosis was 0·7 per 100 child-years (or 700 per 100 000 population per year) and cumulative hazard was 0·02 over 3 years, both of which are high for this age group. Second, the proportion of children with a positive tuberculin conversion was also high, suggesting that many children in this setting acquire infection in the first 2–3 years of life, embodying a large pool at high risk for progressive disease. We found that the hazard of disease progression after primary infection was approximately 0·40 in the first year of life, reducing to less than 0·10 by age 2 years, similar to estimates from historical studies.^19^ Most children who had a tuberculin skin test conversion in our sample did not have documented tuberculosis exposure from their mother or other household members, which suggests a large degree of undiagnosed adult tuberculosis in this high-burden setting or, alternatively, that transmission might occur outside of a known, household source case.^20^, ^21^

We also identified previously unknown risk factors for paediatric tuberculosis infection and disease. To our knowledge, this is the first community-based prospective study to investigate a relationship between lower respiratory tract infection and tuberculosis in infants and young children. A systematic review^22^ of studies with children younger than 5 years with a severe lower respiratory tract infection found high rates of incident tuberculosis ranging from 1% to 23% (5–8% among culture-confirmed cases of tuberculosis). These studies were hospital-based and are thus unlikely to be representative of the community at large because they included children with especially severe disease, a large proportion of whom had HIV infection.^22^, ^23^ These studies did not include control groups of infants without lower respiratory tract infections and therefore it was not possible to estimate an association between the two diseases. In our cohort, we found an association between tuberculosis and lower respiratory tract infection, both of which are highly endemic in South Africa.^15^ The association was robust to varied definitions of tuberculosis diagnosis, including microbiologically confirmed tuberculosis. The observed association between lower respiratory tract infection and tuberculosis, dose response for recurrent lower respiratory tract infection, and persistence of the association despite use of highly sensitive or specific diagnostic testing all suggest that these two conditions might be risk factors for each other. There might also be common risk factors for both conditions in this setting.^15^ The association could have a biological basis or might be related to access to health-care services after presentation of acute lower respiratory tract infection. However, when tuberculosis was diagnosed first and lower respiratory tract infection subsequently, an intrinsic or immunological susceptibility could be a contributing factor. Further study of the possible mechanisms underlying the association between tuberculosis and lower respiratory tract infections is needed. Regardless of the cause, there are important practical implications: integration of disease control measures—eg, screening for tuberculosis infection and disease when infants present to primary care clinics with lower respiratory tract infections—might provide an opportunity to maximise programmatic efficiency in settings where both diseases are endemic.

The increased risk for tuberculin skin test conversion in boys in our study might be explained by immunological or test-specific differences between sexes.^24^, ^25^ Infants of mothers who smoked during pregnancy had 69% greater risk of tuberculosis during follow-up than did unexposed infants. An association between passive smoking and tuberculosis-related outcomes has been shown in adults^26^ and young children.^27^ However, an association between childhood tuberculosis and maternal smoking during pregnancy is new; tobacco smoke exposure might impair neonatal immunological responses,^28^ or this observed relationship might be caused by postnatal smoke exposure. Tuberculin skin test conversion was associated with diagnosed tuberculosis. Pulmonary tuberculosis is a severe, acute disease in infants younger than 3 years, and we have shown that a large proportion of children who had tuberculin conversion rapidly progressed to disease.^19^, ^29^

Isoniazid preventive therapy was effective in preventing tuberculosis. This finding supports previous findings^30^, ^31^ that preventive therapy is one of the most effective interventions to avert progressive disease in hyperendemic settings. Follow-up of these children into the school years will allow us to assess whether preventive therapy provides long-term protection in young children living in such a high-burden setting. It is concerning that of the 147 children with a positive tuberculin skin test who were referred to local clinics for isoniazid preventive therapy, more than 70% were not prescribed isoniazid. This represents a missed opportunity for tuberculosis prevention. Strengthening of current prevention programmes to promote adherence to prophylaxis and development of new strategies for prevention are urgently needed.

Limitations of our study include challenges in the interpretation of longitudinal tuberculin skin test results in a high tuberculosis incidence area. Boosting through BCG vaccination or repeated skin tests could have led to false-positive conversion results. To address this issue, any infant with a positive skin test reaction of any size did not have a repeat skin test, and we acknowledge that dealing with boosting in this manner might have resulted in overestimating or underestimating overall conversion rates. Additionally, tuberculin skin tests are one of several criteria used in clinical diagnosis, potentially inflating disease rates due to tuberculin skin test conversion. Infants with positive skin tests might have been more likely to receive microbiological testing, potentially leading to ascertainment bias. However, in our study sample, most microbiologically confirmed cases were symptomatic and therefore this is doubtful. Additionally, in our tuberculin skin test conversion analysis, informative censoring might have been possible for children diagnosed with recurrent lower respiratory tract infection since they are more likely to receive multiple tuberculin skin tests and thus be censored because of boosting. For all other subgroups and for our disease outcome, censoring in the survival analysis was non-informative. The results might not be generalisable to communities in settings of low tuberculosis prevalence. However, tuberculosis prevalence is high in many African and low-income countries; furthermore, the inclusion of two heterogeneous communities in our study, with risk factors such as poor nutrition or poverty, which are common in many communities in Africa, make these results generalisable to many areas of high tuberculosis prevalence. Lastly, we used self-reported smoking status from mothers. This can be subject to social desirability biases, which, if present, would probably bias the association between maternal smoking and tuberculosis towards the null. Furthermore, maternal self-reported smoking during pregnancy in this cohort was strongly correlated with high urine cotinine levels in both mothers and newborn infants,^32^ suggesting that this bias is unlikely.

In conclusion, we found high incidences of *M tuberculosis* infection and disease in this South African birth cohort, indicating that much greater focus should be placed on the first years of life as a period of high transmission burden and clinical tuberculosis expression. These results support the need for multifaceted interventions, such as wide-scale preventive therapy and integration of control programmes for lower respiratory tract infections and tuberculosis, beginning early in life with the goal of interrupting transmission and preventing progressive disease in paediatric populations living in endemic settings such as South Africa.
