# Supplementary material for: Tuberculin skin test conversion and primary progressive tuberculosis disease in the first 5 years of life: a birth cohort study from Cape Town, South Africa
Source: Lancet Child Adolesc Health. 2018 Jan;2(1):46–55. doi: 10.1016/S2352-4642(17)30149-9 (PMC5810304; doi:10.1016/S2352-4642(17)30149-9)
Supplement: Supplementary appendix [file mmc1.pdf]

THE LANCET
Child & Adolescent Health

Supplementary appendix

This appendix formed part of the original submission and has been peer reviewed. We post it as supplied by the authors.

Supplement to: Martinez L, le Roux DM, Barnett W, Stadler A, Nicol MP, Zar HJ. Tuberculin skin test conversion and primary progressive tuberculosis disease in the first 5 years of life: a birth cohort study from Cape Town, South Africa. *Lancet Child Adolesc Health* 2017; published online Nov 17. http://dx.doi.org/10.1016/S2352-4642(17)30149-9.

SUPPLEMENTARY APPENDIX.

Tuberculin Skin Test Conversion and
Primary Progressive Tuberculosis Disease in the First Five Years of Life:
A Birth Cohort Study from Cape Town, South Africa

Leonardo Martinez[1,2], David M. Le Roux[3], Whitney Barnett[3],
Attie Stadler[3], Mark P. Nicol[4], Heather J. Zar[3]

[1] Stanford University, School of Medicine, Division of Infectious Diseases and Geographic Medicine, Stanford, California, United States;
[2]Department of Epidemiology and Biostatistics, College of Public Health, University of Georgia, Athens, Georgia, United States;
[3]Department of Paediatrics and Child Health, Red Cross War Memorial Children's Hospital and MRC Unit on Child and Adolescent Health, University of Cape Town, Cape Town, South Africa;
[4]Division of Medical Microbiology, University of Cape Town, Cape Town, South Africa

TABLE OF CONTENTS.

1. ADDITIONAL METHODOLOGICAL INFORMATION.
2. SUPPLEMENTARY TABLES.

Supplementary Table 1. Comparison of infant, maternal, and household characteristics of enrolled participants included and not included in the main analysis (N=1143).

Supplementary Table 2. Relationship between a lower respiratory tract infection and tuberculosis disease expressed by hazard ratios and 95% confidence intervals, stratified by the method of tuberculosis diagnosis of the infant.

Supplementary Table 3. Cumulative hazard rate for tuberculin skin test conversion, all diagnosed tuberculosis, microbiologically-confirmed or radiographically diagnosed tuberculosis, and microbiologically-confirmed tuberculosis stratified by infant age.

Supplementary Table 4. Age at tuberculin skin test administration and the size of the skin test induration

Supplementary Table 5. Tuberculosis-related outcomes in children and maternal smoking during pregnancy, restricted to the TC Newman site.

Supplementary Table 6. Test of proportional hazards assumption for variables included in Cox Models of outcomes of tuberculin skin test conversion and diagnosed tuberculosis.

Supplementary Table 7. Clinical and maternal characteristics children with HIV-infection and diagnosed with tuberculosis meningitis included in the study.

3. SUPPLEMENTARY FIGURES.

Supplementary Figure 1. Distribution of tuberculin skin test induration reactions from the first test taken from 915 children in the Drakenstein Child Health Study.

Supplementary Figure 2. Cumulative hazards curves for (A) tuberculin skin test conversion, (B) diagnosed tuberculosis disease, (C), microbiologically or radiographically diagnosed tuberculosis, and (D) microbiologically-confirmed tuberculosis only, Drakenstein Birth Cohort Study.

Supplementary Figure 3. Difference in days between lower respiratory tract infection and tuberculosis diagnoses among all pediatric tuberculosis cases

Supplementary Figure 4. Difference in days† between lower respiratory tract infection and tuberculosis diagnoses in pediatric tuberculosis cases diagnosed microbiologically-confirmed

Supplementary Figure 5. Difference in days† between lower respiratory tract infection and tuberculosis diagnoses in pediatric tuberculosis cases diagnosed either radiographically or microbiologically.

Supplementary Figure 6. Difference in days† between lower respiratory tract infection and tuberculosis diagnoses in all diagnosed tuberculosis cases

ADDITIONAL METHODOLOGICAL INFORMATION.

Nutritional status.

We derived *Z*-scores from World Health Organization child growth standards at birth and at every follow-up visit; we used the median of all the weight-for-age *Z* scores for each child to summarize nutrition status over the duration of follow-up. Children were considered severely underweight or stunted if weight-for-age and length-for-age *Z* scores were less than –2.

Diagnosis of Lower Respiratory Tract Infections.

An active surveillance system was established to detect participants with lower respiratory tract infections or severe lower respiratory tract infections. Study staff undertook surveillance for lower respiratory tract infections at primary care clinics and at Paarl Hospital on weekdays during working hours (0800–1600 h); surveillance continued at Paarl Hospital after hours and over weekends when clinics were closed. If a participant attended a primary health-care clinic or the emergency unit at Paarl Hospital, the healthcare provider could contact the study nurse on the 24-hour mobile phone number. Mothers were given the mobile phone number to enable them to contact the study nurse on call if their child became ill. The study doctor provided regular training for all primary health-care nurses in the accurate assessment of symptoms and signs in young children, and to ensure birth cohort participants were referred to the research study site when lower respiratory tract infections or severe lower respiratory tract infections were diagnosed. Study nurses had regular competency assessments. As the mothers were interviewed frequently through their child's first year of life, and study staff always enquired about previous respiratory events, it was possible to retrospectively capture lower respiratory tract infection events occurring at other facilities or outside the area; information was obtained by review of medical records at the admitting facility.

Specifically, lower respiratory tract infections were diagnosed in children with cough or difficulty breathing and age-specific tachypnea (≥50 breaths per min for children aged 2–12 months) or if the child had lower chest wall indrawing. The treating doctor made the decision to admit a child to hospital; indications included severe lower respiratory tract infections, hypoxia (oxygen saturation of ≤92% in room air), or poor social circumstances in which ambulatory treatment was not feasible. Severe lower respiratory tract infections were diagnosed in children younger than 2 months with tachypnoea (>60 breaths per min) or lower chest wall indrawing, or in children of any age if the child had a general danger sign. Admission of the infant to the hospital was made by the attending clinician and indications for hospital admission included severe lower respiratory tract infections, hypoxia, or infeasibility of ambulatory treatment.

Supplementary Table 1. Comparison of infant, maternal, and household characteristics of enrolled participants included and not included in the main analysis (N=1143).

Variable	Participants included in main analysis [n (%)]*	Participants not included in main analysis [n (%)]*	All Enrolled Participants [n (%)]	*P* Value†
**Infant characteristics**				
N	915 (80.1)	228 (20.0)	1143 (100)	-
Male	468 (51.2)	121 (53.3)	589 (51.6)	0.561
Median birthweight, kilograms (IQR)	3.1 (2.7, 3.4)	3.1 (2.7, 3.4)	3.1 (2.7, 3.4)	0.756
Low birthweight, <2.5 kilograms	135 (14.8)	39 (17.2)	174 (15.2)	0.363
Median gestational age, weeks (IQR)	39 (38, 40)	39 (37, 40)	39 (37, 40)	0.222
Preterm birth, <37 weeks	147 (16.1)	43 (19.2)	190 (16.7)	0.260
Clinic				0.115
Mbekweni	495 (54.1)	137 (60.0)	632 (55.3)	
TC Newman	420 (45.9)	91 (40.0)	511 (44.8)	
Lower Respiratory Tract Infection	440 (48.1)	24 (10.5)	464 (40.6)	<0.01
HIV-exposed	201 (22.0)	48 (21.2)	249 (21.8)	0.788
HIV-positive	2 (0.2)	0 (0)	2 (0.2)	0.481
Median weight for age z-score (IQR)	-0.54 (-1.95, 0.10)	-0.55 (-1.26, -0.02)	-0.55 (-1.31, 0.07)	0.500
Weight for age z-score‡				0.452
Underweight, <-2	84 (9.2)	16 (7.2)	100 (8.8)	
Normal weight, between -2 and 2	812 (89.1)	203 (91.9)	1015 (89.7)	
Overweight, >2	15 (1.7)	2 (0.9)	17 (1.5)	
Received Isoniazid Preventive Therapy	61 (6.7)	14 (6.2)	75 (6.6)	0.810
**Maternal characteristics**				
Median age, years (IQR)	26.3 (22.1, 31.0)	24.3 (21.5, 28.4)	25.8 (22.0, 30.8)	0.002
Married or cohabitating	366 (40.0)	94 (41.6)	460 (40.3)	0.662
TB treatment during pregnancy	41 (4.5)	7 (3.1)	48 (4.2)	0.460
Ever diagnosed with tuberculosis¥	38 (4.2)	3 (1.3)	41 (3.6)	0.044
Current smoker††	218 (24.0)	44 (19.6)	262 (23.1)	0.161
Maternal education				0.023
Primary school only	73 (8.0)	13 (5.7)	86 (7.5)	
Some secondary school	500 (54.6)	107 (47.1)	607 (53.2)	
Finished secondary school	342 (37.4)	107 (47.1)	449 (39.3)	
Formal employment	243 (26.6)	65 (28.6)	308 (27.0)	0.528
**Household Characteristics**				
Socioeconomic status, quartile				0.004
Lowest	233 (25.7)	43 (19.1)	276 (24.4)	
Moderate low	242 (26.7)	47 (20.9)	289 (25.5)	
Moderate high	226 (24.9)	61 (27.1)	287 (25.4)	
Highest	206 (22.7)	74 (32.9)	280 (24.7)	
Household income, rand per month				0.04

<1000	359 (39.2)	71 (31.3)	430 (37.7)	
1000–5000	442 (48.3)	117 (51.5)	559 (49.0)	
>5000	114 (12.5)	38 (17.2)	153 (13.4)	
Informal housing	326 (35.6)	97 (42.7)	423 (37.0)	0.05
Crowding, persons/household (IQR)	4 (3, 6)	4 (3, 6)	4 (3, 6)	0.073
Crowding, persons/household				0.310
3 or fewer	297 (32.5)	86 (37.9)	383 (33.6)	
4 or 5	313 (34.3)	72 (31.7)	385 (33.8)	
More than 5	303 (33.2)	69 (30.4)	372 (32.6)	
Infants <5 years of age/household				0.593
None	563 (61.5)	147 (64.8)	710 (62.2)	
One	291 (31.8)	68 (30.0)	359 (31.4)	
More than one	61 (6.7)	12 (5.3)	73 (6.4)	
TB patient in household in past year	128 (14.1)	30 (13.4)	158 (14.0)	0.781

Abbreviations: TB, tuberculosis. IQR, interquartile range. HIV, Human immunodeficiency virus.
† We used Pearson chi-square and Fisher exact tests as appropriate to derive P values for categorical variables. For continuous variables, we used Wilcoxon rank sum tests for comparison of two-sample medians.
*Percentages refer to within–characteristic column totals among participants within each clinic and in entire study. Percentages may not total 100% because within-column percentages were rounded to the nearest integer.
‡ We derived *Z* scores from World Health Organization child growth standards at birth and at every follow-up visit; we used the median of all the weight-for-age *Z* scores for each child to summarize nutrition status over the duration of follow-up.
¥ This row represents mother that were ever diagnosed with tuberculosis prior to pregnancy.
†† This refers to self-reported smoking status. This was highly correlated with maternal cotinine levels.

Supplementary Table 2. Relationship between a lower respiratory tract infection and tuberculosis disease expressed by hazard ratios† and 95% confidence intervals, stratified by the method of tuberculosis diagnosis of the infant.

Variable	All diagnosed tuberculosis disease	Microbiologically-confirmed or radiographically-suggestive tuberculosis	Microbiologically-confirmed tuberculosis
Any LRTI	2.13 (1.36, 3.32), 0.001	2.83 (1.52, 5.28), 0.001	9.72 (2.24, 42.08), 0.002
Number of LRTI events			
0	1 (Referent)	1 (Referent)	1 (Referent)
1	1.85 (1.13, 3.00), 0.014	2.29 (1.16, 4.54), 0.017	7.19 (1.55, 33.30), 0.012
2	2.28 (1.11, 4.66), 0.025	3.21 (1.29, 7.97), 0.012	8.94 (1.49, 53.69), 0.017
3	4.05 (1.68, 9.74), 0.002	4.80 (1.37, 16.74), 0.014	22.50 (3.16, 160.34), 0.002
4	3.35 (0.80, 14.04), 0.098	6.61 (1.50, 29.17), 0.013	39.98 (5.60, 285.55), <0.01
5	9.29 (2.20, 39.19), 0.002	28.22 (6.23, 127.71), <0.01	117.45 (9.93, 1389.52), <0.01
6	UNDEFINED	UNDEFINED	UNDEFINED
7	UNDEFINED	UNDEFINED	UNDEFINED
LRTI severity			
None	1 (Referent)	1 (Referent)	1 (Referent)
Non-severe	2.21 (1.40 – 3.51), 0.001	2.94 (1.55 – 5.59), 0.001	10.90 (2.49 – 47.68), 0.002
Severe	1.77 (0.84 – 3.74), 0.132	2.41 (0.93 – 6.28), 0.072	5.36 (0.75 – 38.12), 0.093
LRTI care			
None	1 (Referent)	1 (Referent)	1 (Referent)
Ambulatory	1.90 (1.18 – 3.06), 0.008	2.42 (1.24 – 4.70), 0.009	8.65 (1.93 – 38.35), 0.005
Hospitalized	3.00 (1.63 – 5.50), <0.01	4.40 (2.00 – 9.70), <0.01	13.82 (2.68 – 71.24), 0.002

Abbreviations. LRTI, lower respiratory tract infection.
†We adjusted all models for the site of enrollment.

Supplementary Table 3. Cumulative hazard rate for tuberculin skin test conversion╪, all diagnosed tuberculosis¥, microbiologically-confirmed or radiographically diagnosed tuberculosis†, and microbiologically-confirmed tuberculosis‡ stratified by infant age.

Infant age	Tuberculin skin conversion (95% CI)	All diagnosed tuberculosis disease (95% CI)	Microbiologically-confirmed or radiographically diagnosed tuberculosis (95% CI)	Microbiologically-confirmed tuberculosis (95% CI)
**Cumulative Hazard**				
6 months	8.2 (6.4, 10.4)	2.53 (1.68, 3.81)	0.99 (0.51, 1.90)	0.66 (0.30, 1.46)
12 months	16.5 (13.7, 19.9)	6.16 (4.73, 8.02)	2.81 (1.90, 4.15)	1.57 (0.93, 2.65)
24 months	21.6 (18.0, 26.0)	8.40 (6.69, 10.55)	4.46 (3.26, 6.11)	1.80 (1.10, 2.94)
36 months	36.5 (28.9, 46.2)	10.09 (8.12, 12.54)	5.56 (4.15, 7.44)	2.27 (1.44, 3.58)
48 months	–	10.35 (8.33, 12.87)	6.13 (4.56, 8.24)	2.27 (1.44, 3.58)
60 months	–	10.35 (8.33, 12.87)	6.13 (4.56, 8.24)	2.27 (1.44, 3.58)
All ages, rate per year	11.8 (10.0, 13.8)	2.94 (2.38, 3.68)	1.64 (1.23, 2.20)	0.66 (0.41, 1.04)

╪Tuberculin conversion was defined as an induration reaction of greater than or equal to 10-millimeters – children with a reactive skin test <10 millimeters were not given another test at routine visits and were censored. Overall, 147 children tuberculin converted their skin test.

¥ Diagnosed tuberculosis included any type of diagnosis (clinical, radiographical, microbiological). Overall, 81 children had diagnosed tuberculosis.

†Microbiologically-confirmed or radiographically diagnosed tuberculosis included participants with any diagnosis from either a positive microbiological or radiographical test. Overall, 45 children had Microbiologically or radiographically diagnosed tuberculosis.

‡Microbiologically confirmed tuberculosis included participants with a positive Xpert MTB/RIF, sputum culture, or sputum smear test result. Overall, 18 children had microbiologically confirmed tuberculosis.

Supplementary Table 4. Age at tuberculin skin test administration and the size of the skin test induration

Age at TST administration	0mm [n(%)]	0-4 mm [n(%)]	5-9mm [n(%)]	10-14mm [n(%)]	≥15mm [n(%)]	Row Total, N
6 months or below	361 (48.1)	153 (20.4)	97 (12.9)	104 (13.9)	35 (4.7)	750
7-12 months	222 (76.3)	24 (8.2)	20 (6.9)	14 (4.8)	11 (3.8)	291
>12 months to 24 months	286 (85.6)	24 (7.2)	7 (2.1)	5 (1.5)	12 (3.6)	334
>24 months	194 (91.9)	4 (1.9)	1 (0.5)	4 (1.9)	8 (3.8)	211

Supplementary Table 5. Tuberculosis-related outcomes in children and maternal smoking during pregnancy, restricted to the TC Newman site‡.

Variable	Outcome Events	No. Participants	Child-years of observation	Incidence per 100 Child-years (95% CI)	Hazard Ratio (95% CI)	Adjusted Hazard Ratio (95% CI)
**Tuberculin Conversion**						
All participants	76	420	566.2	13.4 (10.7, 16.8)	–	–
Maternal smoking during pregnancy†						
No	37	222	303.7	12.2 (8.8, 16.8)	1 (Referent)	1 (Referent)
Yes	38	195	259.3	14.7 (10.7, 20.1)	1.20 (0.76, 1.88)	1.16 (0.74, 1.83)
**Diagnosed Tuberculosis**						
All participants	55	420	1273.8	4.3 (3.3, 5.6)	–	–
Maternal smoking during pregnancy†						
No	23	222	704.3	3.3 (2.2, 4.9)	1 (Referent)	1 (Referent)
Yes	31	195	561.9	5.5 (3.9, 7.8)	1.64 (0.95, 2.81)	1.66 (0.96, 2.86)

† This refers to self-reported smoking status at the baseline study visit. Self-reported smoking was highly correlated with maternal and infant urine cotinine levels.

‡The multivariable model for tuberculosis conversion was adjusted for male sex, maternal smoking during pregnancy, the number of children <5 years old in the household, and the age at which the first tuberculin skin test was performed. The multivariable model for diagnosed tuberculosis was adjusted for male sex, isoniazid preventive therapy, tuberculin skin test conversion, a lower respiratory tract infection, and maternal smoking during pregnancy.

Supplementary Table 6. Table. Test of proportional hazards assumption for variables included in Cox Models of outcomes of tuberculin skin test conversion and diagnosed tuberculosis.

Variable	Proportional hazards assumption	
	Tuberculin skin test conversion	Diagnosed Tuberculosis
**Child characteristics**		
Sex	Not violated	Not violated
Birthweight, kilograms	Not violated	Not violated
Gestational age, weeks	Not violated	Not violated
Lower respiratory tract infection	Not violated	Not violated
HIV-positive	Not violated	Not violated
Weight for age Z-score	Not violated	Not violated
Breastfeeding	Not violated	Not violated
Isoniazid Preventive Therapy	Not violated	Not violated
**Maternal characteristics**		
Age, years	Not violated	Not violated
TB treatment in pregnancy	Not violated	Not violated
Prior TB diagnosis	Not violated	Not violated
Maternal smoking during pregnancy	Not violated	Not violated
Maternal education	Not violated	Not violated
**Household Characteristics**		
Socioeconomic status, quartile	1 strata violated, P=0.03	1 strata violated, P=0.04
Clinic	Not violated	Not violated
Household income, rand per month	Not violated	Not violated
Crowding, persons per household	Not violated	1 strata violated, P=0.03
Children under 5 yo per household	Not violated	Not violated
TB patient in household in past year	Not violated	Not violated

Supplementary Table 7. Infant and maternal clinical characteristics of children with HIV-infection and diagnosed with tuberculosis meningitis included in the study.

Infant	Infant HIV-status	Diagnosed tuberculosis, type	Maternal HIV-status	TB patient in household in past year‡	Preventive therapy given	Maternal TB treatment during pregnancy	Mother diagnosed with TB prior to pregnancy	Breastfed
1	Positive	Yes, Pulmonary	Positive	No	No	No	No	Yes
2	Positive	No	Positive	No	No	No	No	Yes
3	Negative	Yes, Meningitis	Positive	Yes	No	Yes	No	Yes

Abbreviations. TB, tuberculosis.
‡ Household tuberculosis exposure in the year before the primary study visit

Supplementary Figure 1. Distribution of tuberculin skin test induration reactions from the first test taken from 915 children in the Drakenstein Child Health Study†

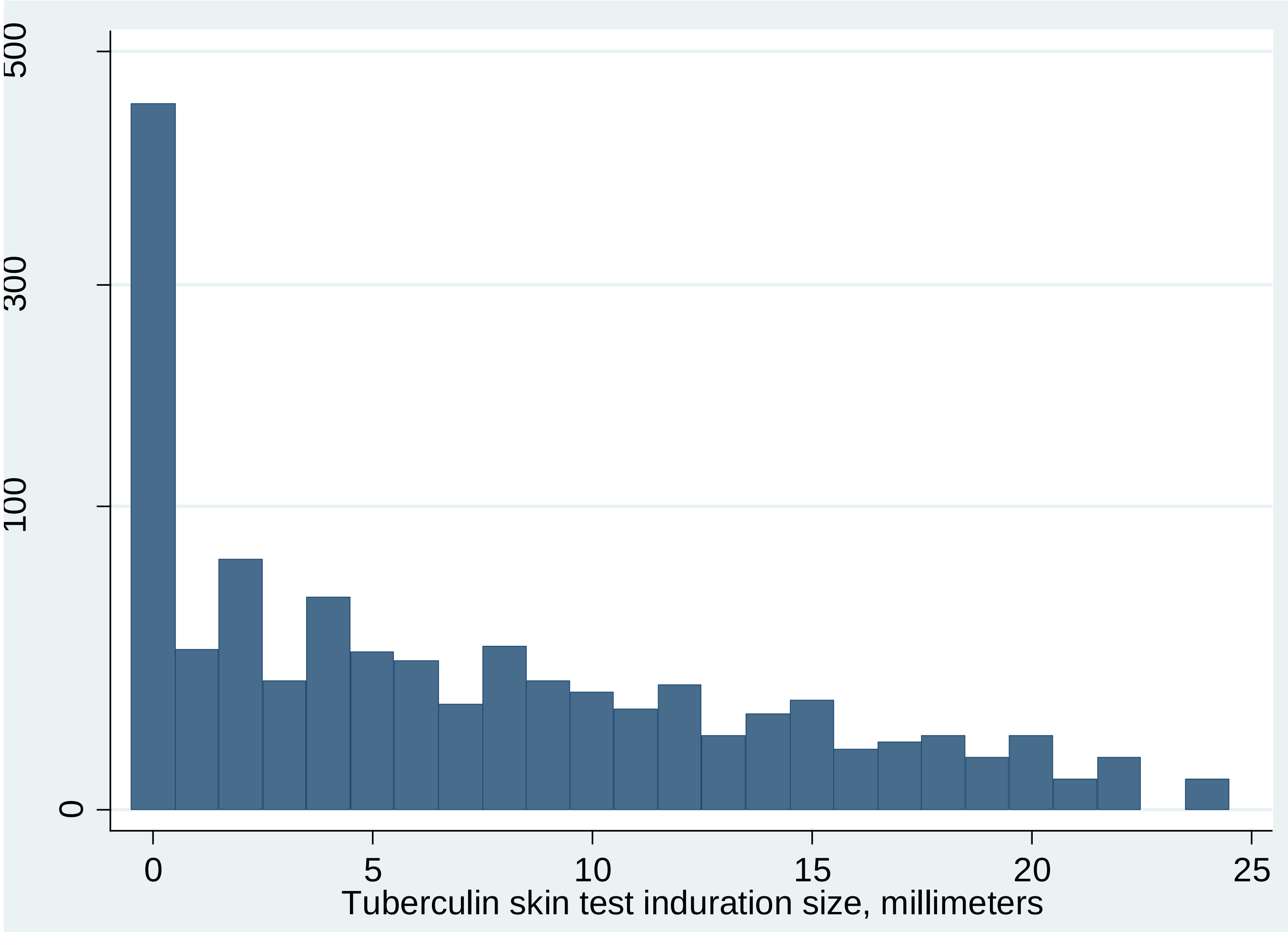

† The y-axis is set on a square root scale due to the large number of participants with null induration reactions. Therefore, the spaces amongst the y-axis may not be proportionate. A tuberculin skin test conversion was defined as a skin test induration of 10 millimeters or greater.

Supplementary Figure 2. Cumulative hazards curves for (A) tuberculin skin test conversion, (B) diagnosed tuberculosis disease, (C), microbiologically or radiographically diagnosed tuberculosis, and (D) microbiologically-confirmed tuberculosis only, Drakenstein Birth Cohort Study.

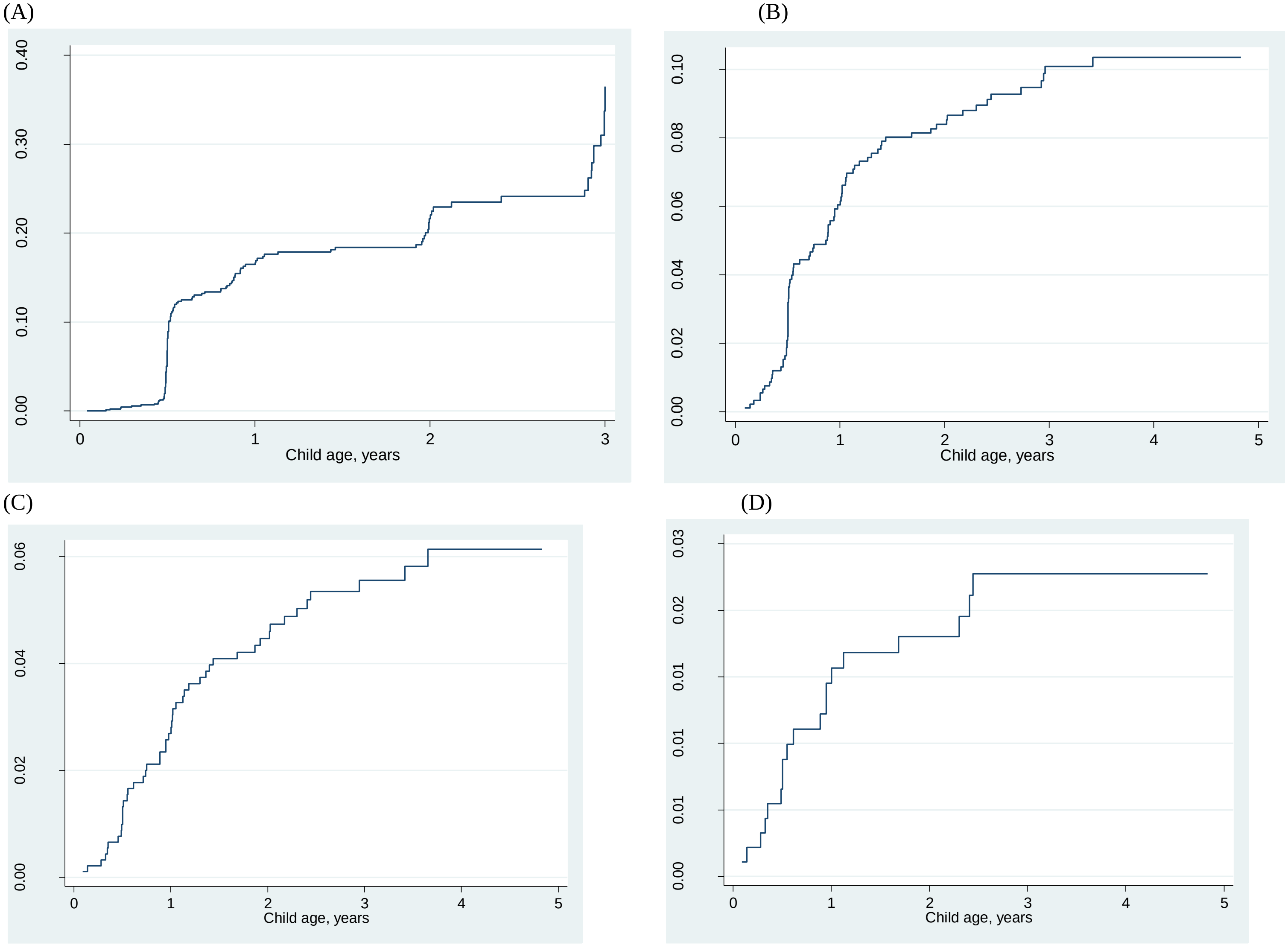

Supplementary Figure 3. Difference in days between lower respiratory tract infection and tuberculosis diagnoses among 55 children with both diagnoses.

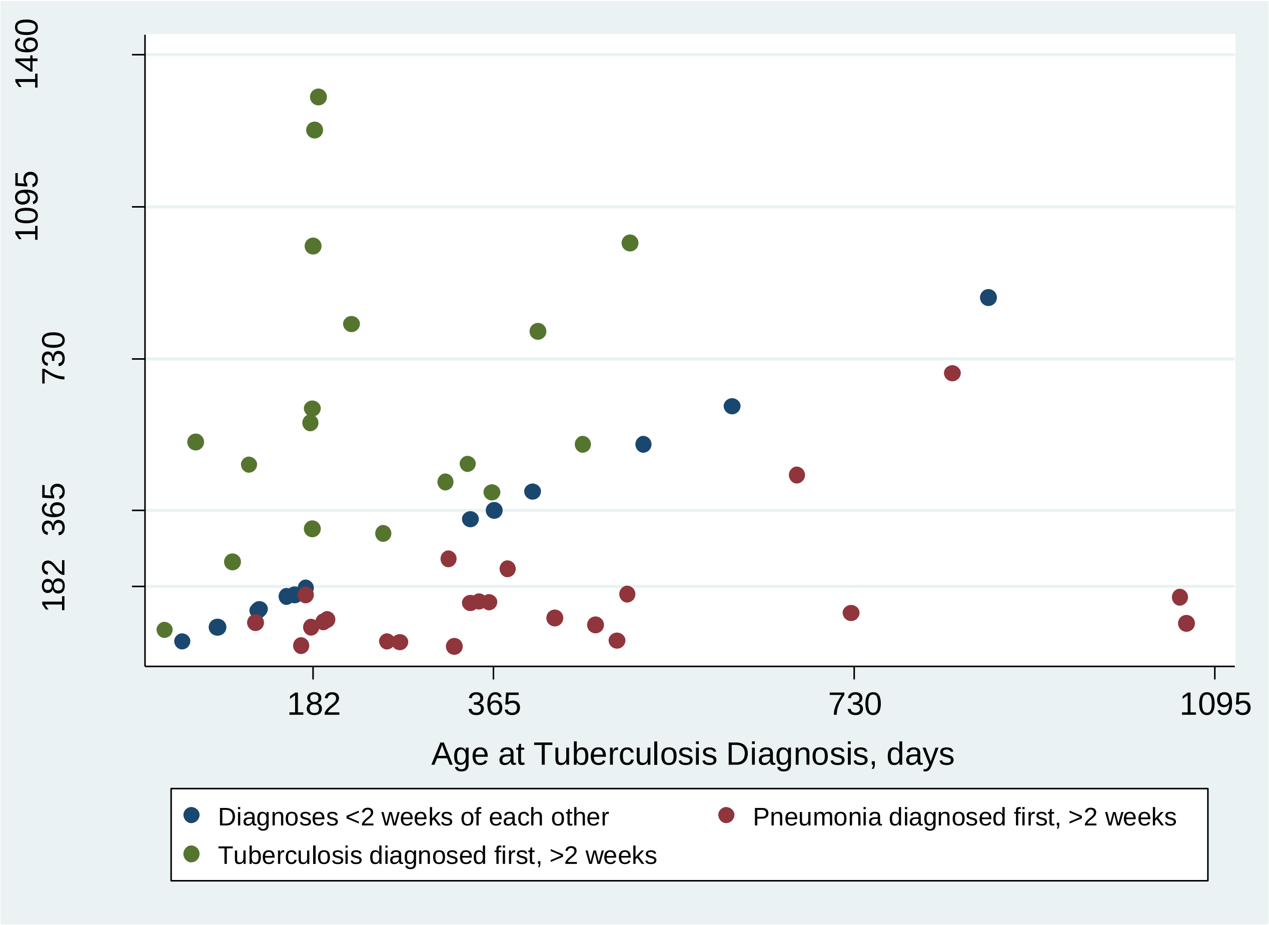

Supplementary Figure 4. Difference in days† between lower respiratory tract infection and tuberculosis diagnoses in pediatric tuberculosis cases diagnosed microbiologically-confirmed

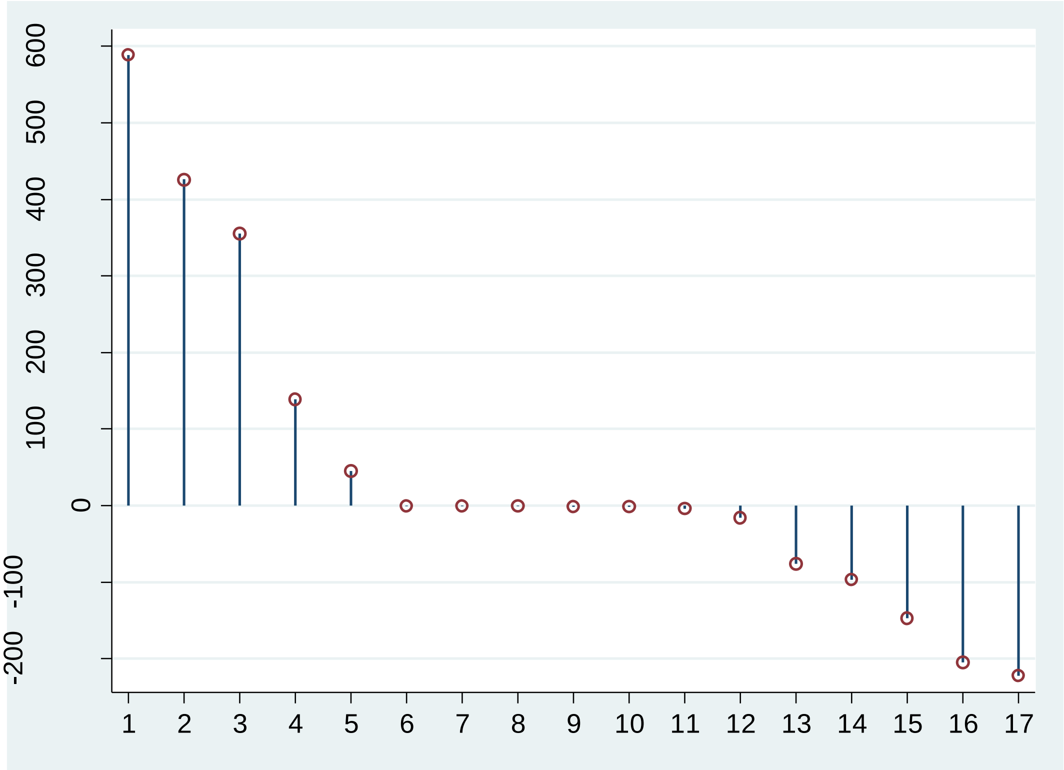

† Positive numbers on the y-axis indicate the tuberculosis diagnosis occurred before the lower respiratory tract infection. Negative numbers on the y-axis indicate the lower respiratory tract infection occurred before the tuberculosis diagnosis. Cases with circles at 0 days indicates both diagnosis occurred at the same time. The lower respiratory tract infection and tuberculosis disease were diagnosed more than two weeks apart from each other in 11 of 17 (64.7%) microbiologically-confirmed cases that also were diagnosed with a lower respiratory tract infection.

Supplementary Figure 5. Difference in days† between lower respiratory tract infection and tuberculosis diagnoses in pediatric tuberculosis cases diagnosed either radiographically or microbiologically.

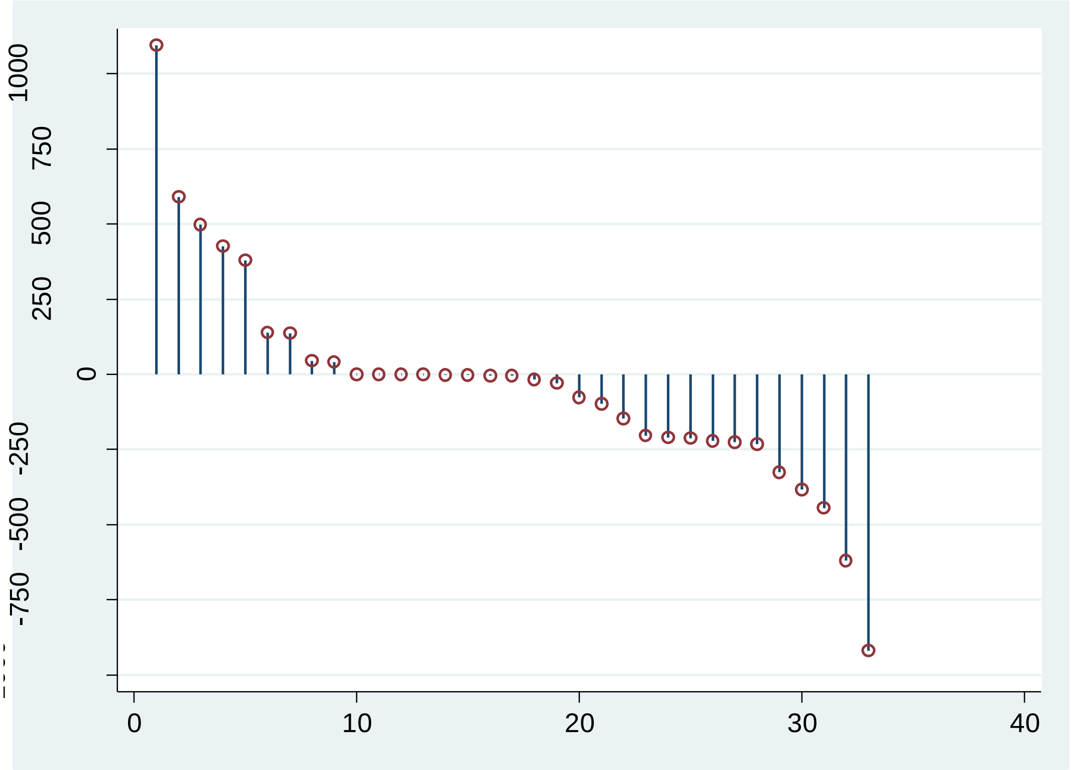

† Each vertical line represents a single child with both lower respiratory tract infection and a tuberculosis diagnoses and the y-axis gives a unique ID number for each child. Positive numbers on the y-axis indicate the tuberculosis diagnosis occurred before the lower respiratory tract infection. Negative numbers indicate the lower respiratory tract infection occurred before the tuberculosis diagnosis. Cases with circles at 0 days indicates both diagnosis occurred at the same time. The lower respiratory tract infection and tuberculosis disease were diagnosed more than two weeks apart from each other in 26 of 34 (76.5%) radiographically suggestive or microbiologically-confirmed cases that also were diagnosed with a lower respiratory tract infection.

Supplementary Figure 6. Difference in days† between lower respiratory tract infection and tuberculosis diagnoses in all diagnosed tuberculosis cases

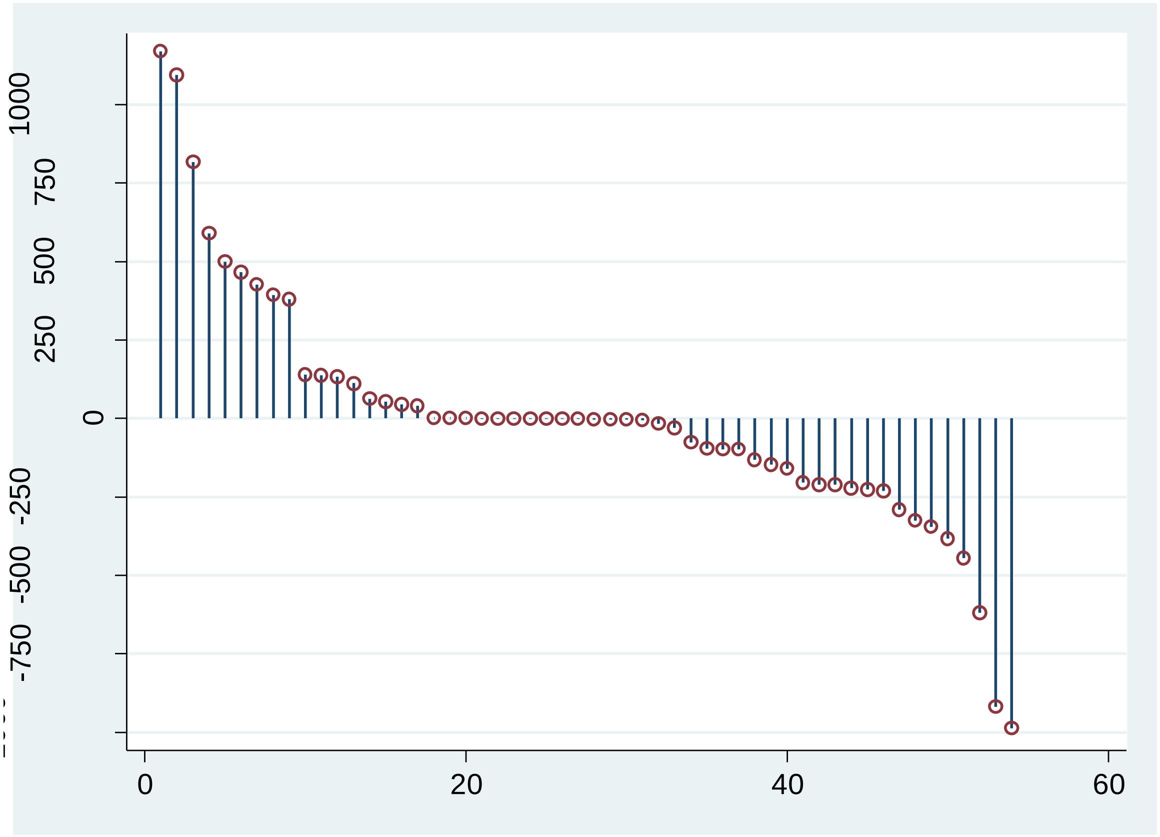

† Each vertical line represents a single child with both lower respiratory tract infection and a tuberculosis diagnoses and the y-axis gives a unique ID number for each child. Positive numbers on the y-axis indicate the tuberculosis diagnosis occurred before the lower respiratory tract infection. Negative numbers on the y-axis indicate the lower respiratory tract infection occurred before the tuberculosis diagnosis. Cases with circles at 0 days indicates both diagnosis occurred at the same time. The lower respiratory tract infection and tuberculosis disease were diagnosed more than two weeks apart from each other in 41 of 55 (74.5%) of all diagnosed tuberculosis cases (clinically, radiographically, or microbiologically diagnosed disease) that also were diagnosed with a lower respiratory tract infection.
